# Investigations of the functional states of dendritic cells under different conditioned microenvironments by Fourier transformed infrared spectroscopy

**DOI:** 10.1186/1475-925X-13-2

**Published:** 2014-01-10

**Authors:** Rong Dong, Jinhua Long, Xiaoli Xu, Chunlin Zhang, Zongyao Wen, Long Li, Weijuan Yao, Zhu Zeng

**Affiliations:** 1 Department of Biomedical Engineering, Guiyang Medical College, Guiyang, Guizhou Province 550004, People’s Republic of China; 2 Department of Cell Biology, Guiyang Medical College, Guiyang, Guizhou Province 550004, People’s Republic of China; 3 Department of Head and Neck, Affiliated Cancer Hospital, Guiyang Medical College, Guiyang, Guizhou Province 550004, People’s Republic of China; 4 Department of Nephrology, Affiliated Hospital of Guiyang Medical College, Guiyang, Guizhou Province 550004, P. R. China; 5 Center of Hemorheology, School of Basic Medical Sciences, Health Science Center of Peking University, Beijing 100083, People’s Republic of China; 6 Department of Biotechnology, Guiyang Medical College, Guiyang, Guizhou Province 550004, P. R. China

**Keywords:** Dendritic cell, Fourier transformed infrared spectroscopy, Functional states of cells, NF-κB

## Abstract

**Background:**

Dendritic cells are potent and specialized antigen presenting cells, which play a crucial role in initiating and amplifying both the innate and adaptive immune responses. The dendritic cell-based vaccination against cancer has been clinically achieved promising successes. But there are still many challenges in its clinical application, especially for how to identify the functional states.

**Methods:**

The CD14^+^ monocytes were isolated from human peripheral blood after plastic adherence and purified to approximately 98% with cocktail immunomagnetic beads. The immature dendritic cells and mature dendritic cells were induced by traditional protocols. The resulting dendritic cells were cocultured with normal cells and cancer cells. The functional state of dendritic cells including immature dendritic cells (imDCs) and mature dendritic cells (mDCs) under different conditioned microenvironments were investigated by Fourier transformed infrared spectroscopy (FTIR) and molecular biological methods.

**Results:**

The results of Fourier transformed infrared spectroscopy showed that the gene transcription activity and energy states of dendritic cells were specifically suppressed by tumor cells (*P* < 0.05 or 0.01). The expression levels of NF-kappa B (NF-κB) in dendritic cells were also specifically inhibited by tumor-derived factors (*P* < 0.05 or 0.01). Moreover, the ratios of absorption intensities of Fourier transformed infrared spectroscopy at given wave numbers were closely correlated with the expression levels of NF-κB (R^2^:0.69 and R^2^:0.81, respectively).

**Conclusion:**

Our results confirmed that the ratios of absorption intensities of Fourier transformed infrared spectroscopy at given wave numbers were positively correlated with the expression levels of NF-κB, suggesting that Fourier transformed infrared spectroscopy technology could be clinically applied to identify the functional states of dendritic cell when performing dendritic cell-based vaccination. It’s significant for the simplification and standardization of dendritic cell-based vaccination clinical preparation protocols.

## Introduction

Dendritic cells (DCs) are potent and specialized antigen-presenting cells with the capabilities of capturing, processing antigens and presenting tumor associated antigens to naive-T cells, therefore inducing tumor-specific immune response [1,2]. DCBV is a promising immunotherapy against cancer, which has been shown to effectively work in mice and human. However, there are still many challenges in clinical application of DCBV, such as antigen selection, ways of antigen loading, pathways and intervals of injection, and identification of the functional state of DCs [3-6]. Many strategies have been focused on the improvement of the efficiency of DCBV [1,5,7], nevertheless, little about identification of functional state of cells. Especially, it was clinically cumbersome for the preparation of DCBV and difficult to determine the functional state of DCs. Therefore, how to measure the functional state of DCs with a convenient, reliable and non-invasive method is practically for DCBV preparation protocols.

FTIR is a spectroscopic technique which is used to obtain an infrared spectrum of absorption, emission, photoconductivity or Raman scattering of solid, liquid or gas [8]. With its simple, rapid, and reliable analysis, it has been widely used in the studies of medicine, e.g. cancer identification [9]. Presently, it is well confirmed that DCs are dysfunctional in cancer-bearing hosts [10-13]. NF-κB is a major transcription factor that regulates genes responsible for both the innate and adaptive immune response. Karin and colleagues found that NF-κB is associated with the immunological functions of mast cells and DCs in the liver, which is shared by many signal pathways. Moreover, the activation of NF-κB in these cells can lead to the expression of cytokines and chemokines [14,15]. RelB is a subunit of NF-κB transcription factor which played a key role in the maturity of DCs [16]. Recent evidences demonstrated that the generation of tolerogenic DCs and immune tolerance were induced by RelB-silence in organ transplantation [17-20]. Several groups suggested that the immunological functions of DCs are deteriorated by tumor through the inhibition of NF-κB function [14,21,22]. Therefore, NF-κB was selected as the indicator of gene transcription of DCs in the present study. In this study, the functional states of DCs at different differentiation stages (imDCs and mDCs) under various conditioned microenvironment were measured by FTIR and molecular biological technologies. The results showed that the gene transcription activity and energy states of DCs were suppressed by tumor cells, moreover, the absorption intensities of FTIR at given wave number were closely correlated with the expression levels of RelB. It laid the foundation for the application of FTIR to the identification of functional states in the DCBV preparation protocol.

## Materials and methods

### Isolation of monocytes and generation of DCs

The DCs were generated from fresh peripheral blood mononuclear cells (PBMCs) of healthy human subject as described by Steinman [23] with minor modification. Details as follows: CD14^+^ monocytes were obtained from peripheral blood after plastic adherence and purified to approximately 98% by elimination of un-mononuclear cells (T-cells, NK cells, B cells, neutrophile cells and dendritic cells) with cocktail immunomagnetic beads (anti-CD3, anti-CD7, anti-CD19, anti-CD56, anti-CD45RA and anti-IgE antibody). The monocytes were cultured in RPMI 1640 medium supplemented with 20% fetal bovine serum (FBS), glutamine, penicillin and streptomycin, plus 150 ng/ml recombinant human granulocyte-macrophage colony stimulating factor (rhGM-CSF) and 100 ng/ml recombinant human interleukin-4 (rhIL-4). After 7 days, collected suspension of cells was imDCs, and 10 ng/ml recombinant human tumor necrosis factor (rhTNF-α) was added and the imDCs were further cultured for another 3 days to obtain mDCs. The phenotypes of DCs were analyzed by staining the cell surface with FITC or PE-conjugated mouse antibodies to human CD11c, CD40, CCR7, CD80, CD83, CD86 and HLA-DR. The viabilities of cells were detected by trypan blue staining.

### Isolation and culture of human umbilical vein endothelial cells (HUVECs)

As one of the normal controls, HUVECs were separated from umbilical cords (provided by the Third Hospital of Peking University) and maintained in M199 medium/20% FBS. The experiments were conducted with strict observance of the moral principles and guidelines for human investigation in China, where the experiments were performed. The cells were identified by their polyangular shape and the presence of factor VIII-related antigen (Santa Cruz Biotechnology Inc., Santa Cruz, USA) in their cytoplasm.

### Cultures of cell lines

Hepatocellular carcinoma cell line Bel7402 (HCC) and normal hepatocyte cell line QSG-7701 (HC) were gifted by Dr. Ye Zhang (Peking University). Cells (2 × 10^6^) were rinsed with phosphate-buffered saline (PBS) and cultivated in RPMI-1640 with 10% fetal bovine serum (FBS) at 37°C, 5% CO_2_ and 95% humidity. Cells were digested every 3 days by trypsinization with 10% trypsin/EDTA. The cells in logarithmic phase were collected to co-cultured with DCs.

### Co-culture of DCs with different cells

The co-culture of imDCs or mDCs (2 × 10^7^) with HUVEC, HCC and HC (2 × 10^6^) were performed in the Transwell system (0.6 μm pores, Corning corporation) for 24 hours, respectively. The HUVEC and HC were as negative control. DCs were seeded on the upper compartment of Transwell chambers, HUVEC, HCC and HC were placed in the lower compartment respectively. These DCs were labeled as imDCs + HUVEC, mDCs + HUVEC, imDCs + HC, mDCs + HC, imDCs + HCC, mDCs + HCC. For blank controls, DCs were cultured in complete medium supplemented with 150 ng/ml rhGM-CSF and 100 ng/ml rhIL-4 and DCs were cultured in RPMI-1640 plus 20% FBS without rhGM-CSF and rhIL-4 were labeled as DCs + nonGF (including imDCs + nonGF and nonGF).

### Treatments of DCs

In order to determine susceptibility of FTIR used in the measurement of cell gene transcription activity, imDCs and mDCs were processed by 5 nM ASA (Santa Cruz Biotechnology Inc., Santa Cruz, USA), a specific inhibitor of NF-κB, for 24 hours and named as imDCs + ASA and mDCs + ASA.

### FTIR measurements

Cells were adjusted to 2 × 10^6^/ml and washed twice with 0.9% NaCl in 1000 RMP centrifugation for 6 min. The supernatant was removed by centrifugation. The cells were transferred to the CaF_2_ crystals at 37°C and left to stand for about 10 min. The water in the cell suspension was evaporated, until the formation of 2 ~ 3 mm film in the window. The crystals were fixed in the sample holder and covered with another CaF_2_ crystal. To measure the background spectrum of a blank group, before each sample measurement by Infrared Spectrometer (ENXUS-470 FT-IR), blank control with 0.9% NaCl was used in the detection of infrared absorption spectra. The parameters of measurement were the scanning range of 400 ~ 4000 cm^-1^, the resolution of 8 cm^-1^, scanning the stack up to 256 times. The data analyses were performed using OMNIC6.0 software. All the spectra were subtracted blank control, and Fourier self-deconvolution, broadband = 56.4, sensitivity enhancement factor = 2.6, in deconvolution spectrum.

### Western blots

Cells were lysed with RIPA buffer (20 mM sodium phosphate, pH 7.4, 150 mM sodium chloride, 1% Triton X-100, 5 mM EDTA, 200 μM phenymethylsulfonyl fluoride, 1 μg/ml aprotinin, 5 μg/ml leupeptin, 1 μg/ml pepstatin and 500 μM Na_3_VO_4_). The protein extracts were electrophoresed on 12% ~ 14% SDS-polyacrylamide gel and transferred onto a nitrocellulose membrane (Invitrogen, USA). After blocking with 5% BSA in 0.1% Tween 20 in PBS, membranes were probed with primary antibodies. Anti-RelB and anti-β-actin antibodies (Sigma) were diluted in blocking buffer and incubated with the blots overnight at 4°C. The bound primary antibodies were probed with a 1:2000 diluted secondary antibody (goat anti-human IgG-HRP antibody) and visualized by the ECL chemiluminescence system (Amersham, USA). The gray values of proteins were measured by Image J (1.45). The expression levels of proteins were normalized to those of corresponding β-actin.

### Statistical analyses

All experiments were performed at least three times. The results were presented as means ± standard deviations (SD). Analysis of variance (ANOVA two-way) of SPSS (11.5) was used for statistical data analyses. Correlation analyses were performed by Excel (2007).

## Results

### Characterizations of imDCs and mDCs

The expression profiles of co-stimulatory molecules, including CD11c, CD40, HLA-DR, CCR7, CD80, CD83 and CD86, were analyzed for both imDCs and mDCs with flow cytometry. The expression levels of these co-stimulatory molecules in mDCs were up-regulated by IL-10, suggesting that imDCs and mDCs were successfully induced [24,25].

### The energy states and gene transcription activity in DCs co-cultured with different cells

**Figure 1:**
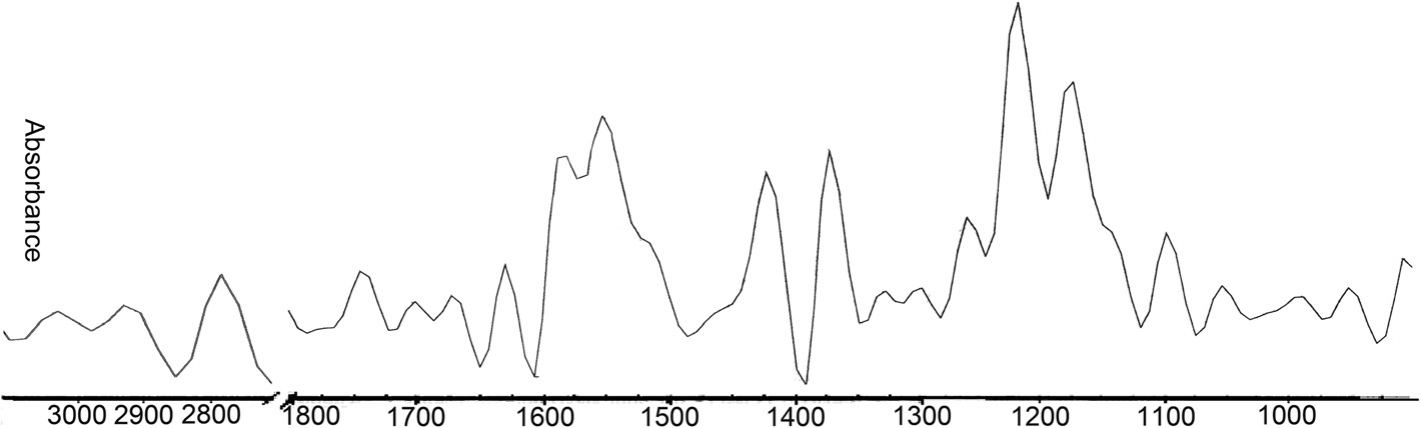
**The schematic diagram of infrared spectrum of the contents of lipids and proteins in cells.** Cells were adjusted to 2 × 10^6^/ml and washed twice with 0.9% NaCl in 1000 RPM centrifugation for 6 min. The supernatant was removed by centrifugation. The cells were transferred to the CaF_2_ crystals at 37°C and left to stand for about 10 min. The water in the cell suspension was evaporated, until the formation of 2 ~ 3 mm film in the window. The crystals were fixed in the sample holder and covered with another CaF_2_ crystal. To measure the background spectrum of a blank group, before each sample measurement by Infrared Spectrometer (ENXUS-470 FT-IR), blank control with 0.9% NaCl was used in the detection of infrared absorption spectra. The parameters of measurement were the scanning range of 400 ~ 4000 cm^-1^, the resolution of 8 cm^-1^, scanning the stack up to 256 times. The data analyses were performed using OMNIC6.0 software. All the spectra were subtracted blank control, and Fourier self-deconvolution, broadband = 56.4, sensitivity enhancement factor = 2.6, in deconvolution spectrum.

According to the results of the Ramesh group [26,27], the absorption intensity ratios of A_1020/A1545_, A_1121_/A_1545_, A_1030_/A_1080_ and A_1030_/A_2924_ respectively correspond to DNA/amide II (relative content of DNA and proteins), RNA/amide II (transcriptional states), glucose/phosphate (metabolic turnover) and glucose/phospholipid (*de onvo* synthesis of phospholipids at the expense of free glucose). All spectra were subjected to Fourier self-deconvolution, and there was no wave number shift in the spectra (Figure 1). The ratios of absorption intensities of A_1020/_A_1545_, A_1121_/A_1545_, A_1030_/A_1080_ and A_1030_/A_2924_ of DCs under different conditioned microenvironments were shown in Table 1, the A_1121_/A_1545_, A_1030_/A_1080_ and A_1030_/A_2924_ of DCs + HCC and DCs + ASA were significantly lower than those of controls (^*^P < 0.05 or ^**^P < 0.01), moreover, there was no difference among DCs + HUVEC, DCs + HC and DCs (*P* > 0.05).

**Table 1:** The ratios of absorption intensity at given wave number in DCs under different conditioned microenvironments (X ± SD)

	**Types of DCs**	**A**_**1020/A1545**_	**A**_**1121**_**/A**_**1545**_	**A**_**1030**_**/A**_**1080**_	**A**_**1030**_**/A**_**2924**_
		**DNA/amide II**	**RNA/amide II**	**Glucose/phospholipid**	**Glucose/phosphate**
DCs	imDCs	3.782 ± 0.016	2.953 ± 0.060	1.274 ± 0.046	0.753 ± 0.033
	mDCs	0.531 ± 0.032	1.219 ± 0.039	0.169 ± 0.022	0.957 ± 0.058
DCs + nonGF	imDCs + nonGF	3.655 ± 0.027	1.822 ± 0.086	1.383 ± 0.031	0.764 ± 0.028
	mDCs + nonGF	0.583 ± 0.026	0.877 ± 0.051	0.423 ± 0.015	0.604 ± 0.022
DCs + HUVEC	imDCs + HUVEC	3.452 ± 0.187	2.769 ± 0.171	1.302 ± 0.054	0.748 ± 0.048
	mDCs + HUVEC	0.549 ± 0.079	1.233 ± 0.089	0.152 ± 0.103	0.933 ± 0.021
DCs + HC	imDCs + HC	3.556 ± 0.045	2.843 ± 0.062	1.266 ± 0.082	0.712 ± 0.067
	mDCs + HC	0.586 ± 0.034	1.176 ± 0.095	0.149 ± 0.061	0.926 ± 0.043
DCs + HCC	imDCs + HCC	3.721 ± 0.027	0.603 ± 0.004^*^	0.382 ± 0.010^*^	0.302 ± 0.017^*^
	mDCs + HCC	0.577 ± 0.027	0.361 ± 0.021^*^	0.423 ± 0.007^*^	0.408 ± 0.023^*^
DCs + ASA	imDCs + ASA	3.718 ± 0.018	0.296 ± 0.021^**^	0.351 ± 0.012^**^	0.281 ± 0.024^**^
	mDCs + ASA	0.488 ± 0.024	0.125 ± 0.009^**^	0.107 ± 0.009^**^	0.386 ± 0.031^**^

imDCs + HCC and mDCs + HCC respectively compared with imDCs and mDCs: ^*^p < 0.05; imDCs + ASA and mDCs + ASA respectively compared with imDCs and mDCs: ^**^p < 0.01.

### The expression levels of RelB were down-regulated in DCs co-cultured with HCCs

**Figure 2:**
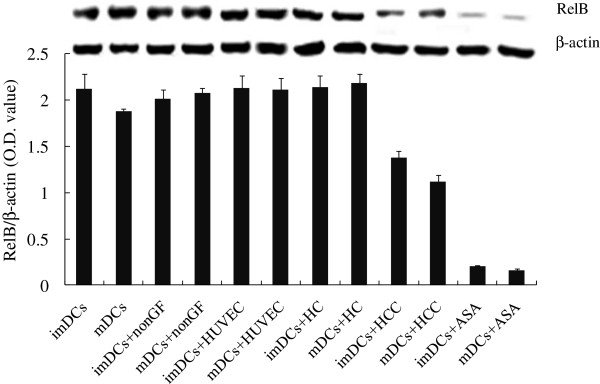
**The expression levels of RelB in DCs under different conditioned microenvironments.** Cells were lysed with RIPA buffer (20 mM sodium phosphate, pH 7.4, 150 mM sodium chloride, 1% Triton X-100, 5 mM EDTA, 200 μM phenymethylsulfonyl fluoride, 1 μg/ml aprotinin, 5 μg/ml leupeptin, 1 μg/ml pepstatin and 500 μM Na_3_VO_4_). The protein extracts were electrophoresed on 12% ~ 14% SDS-polyacrylamide gel and transferred onto a nitrocellulose membrane (Invitrogen, USA). After blocking with 5% BSA in 0.1% Tween 20 in PBS, membranes were probed with primary antibodies. Anti-RelB and anti-β-actin antibodies (Sigma) were diluted in blocking buffer and incubated with the blots overnight at 4°C. The bound primary antibodies were probed with a 1:2000 diluted secondary antibody (goat anti-human IgG-HRP antibody) and visualized by the ECL chemiluminescence system (Amersham, USA). The gray values of proteins were measured by Image J (1.45). The expression levels of proteins were normalized to those of corresponding β-actin. Compared with DCs: ^*^P < 0.05 or ^**^P < 0.01.

RelB is a subunit of NF-κB, whose expression levels are closely associated with the mature state of DCs [28]. As shown in Figure 2, the expression levels of RelB in DCs + HCC (imDCs and mDCs) were markedly inhibited by tumor cells (^*^P < 0.05 or ^**^P < 0.01). Moreover, the expression levels of RelB in cells were significantly down-regulated by NF-κB inhibitor ASA, which were not affected by HUVEC and HC (*P* > 0.05).

### Analyses of linear regression

**Figure 3:**
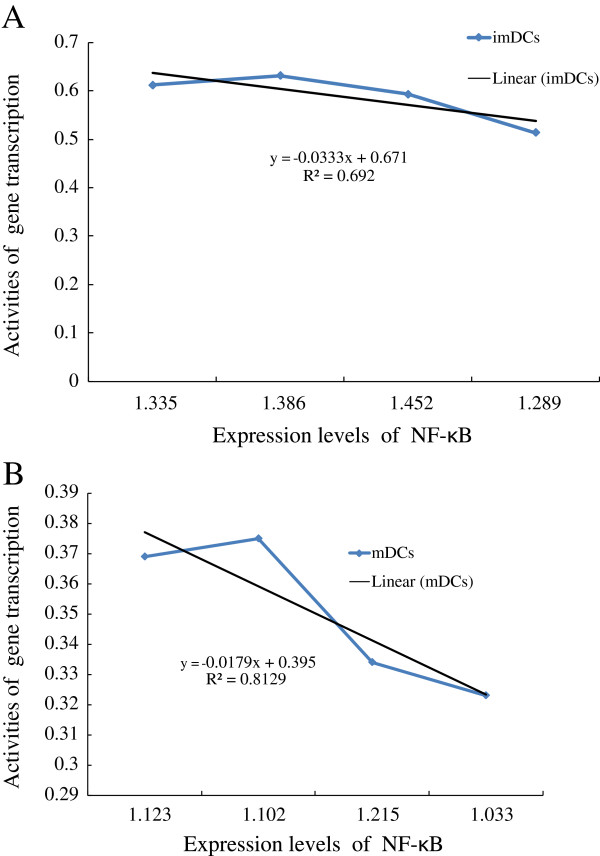
**Analyses of linear regression of the activities gene transcription and the expression levels of NF-κB. A**. imDCs (*R*^*2*^: 0.69), **B**. mDCs (*R*^*2*^: 0.81).

To investigate the correlations between the expression levels of NF-κB in DCs and the ratios of absorption intensities at given wave numbers, the analyses of linear regression were performed. The results (Figure 3) showed that the values of A_1121_/A_1545_ of DCs + HCC (imDCs + HCC and mDCs + HCC) were closely correlated with the expression levels of NF-κB (*R*^*2*^:0.69 and *R*^*2*^:0.81, respectively).

## Discussion

Along with the first DCs-based therapeutic cancer vaccine was approved by the Food and Drug Administration of USA, DCBV have been having a promising future because it was demonstrated feasibilities and safeties [5,29]. Clinically, DCBV has been achieved some successes, despite their induction of tumor-specific T cell responses in many patients and occasional complete tumor regressions, there are still many conundrums, including antigen selection, injection of pathways and intervals, immune escape of cancer, identifications of DCs functional state and so on, need to be overcome [1,5,7,30]. Moreover, the preparation protocols of DCBV also should be simplified and standardized for their better clinical applications. The present study was focused on the measurement of functional states of DCs with FTIR in order to find a convenient, reliable and non-invasive method for DCBV preparation protocol.

The FTIR technique can be applied to investigating the vibration modes of functional radicals in molecules in response to changes in cell structure, conformation and microenvironment. It has become a useful tool for measuring the macromolecular characteristics and biochemical event. Here, the functional states of imDCs and mDCs under different conditioned microenvironments by FTIR spectroscopy and molecular biological methods.

The ratio of A_1121_/A_1545_ corresponds to an RNA/amide, which reflects the gene transcription states of cells. As shown in Table 1, the ratios of A_1121_/A_1545_ of DCs + HCC were significantly lower than those of DCs (^*^P < 0.05) and there were no difference among DCs + HUVEC, DCs + HC and DCs (*P* > 0.05), suggesting that the gene transcription states of DCs were specifically suppressed by HCC, this is consistent with others [14,15,31]. The inhibition of gene transcription state might be associated with the abnormal expressions of some important proteins in DCs leading to the dysfunctional immune responses of the host. Under the tumor microenvironment, NF-κB plays an important role in cancer development and progression because it’s a crucial transcription factor which regulates immune functions [32]. Kuwabara and colleagues found that the NF-κB signaling pathways perform a critical role in CCR7-mediated IL-23 production [33]. The RelB subunit of NF-κB controls DCs maturation and may be therapeutically targeted to manipulate the T cell response in disease. Several groups have reported that RelB promoted DC activation, and RelB-silenced DCs could induce donor-specific hypo-responsiveness and impair immune functions of T cells [28,34]. The tumor microenvironment-derived immunosuppressive cytokines (VEGF, TGF, IL-10 et al.) down-regulate the expression levels of chemokines, adhesion molecule and costimulatory molecules by suppressing expression of NF-κB [35,36]. Therefore, the expression levels of NF-κB in cells were selected as one of the indicators of DCs functional states. To confirm the results of FTIR, DCs were treated with NF-κB inhibitor ASA, the data (Table 1) showed that the ratio of A_1121_/A_1545_ of DCs was indeed markedly decreased by ASA, this was consistent with the data of western blotting (Figure 2). Moreover, the ratios of A_1121_/A_1545_ (Figure 3) were closely correlated with the expression levels of NF-κB (*R*^2^: 0.69 and 0.81). Thus, these results have supported the idea that FTIR could be clinically applied to estimate the functional states of DCs.

The ratios of A_1030_/A_1080_ and A_1030_/A_2924_ respectively respond to glucose/phospholipids and glucose/phosphate, which reflect the energy states of cells. As shown in Table 1, the ratios of A_1030_/A_1080_ and A_1030_/A_2924_ of DCs + HCC were lower than those of DCs (*P* < 0.05) and there were no difference among DCs + HUVEC, DCs + HC and DCs (*P* > 0.05), indicating that the energy states of DCs were also significantly and specifically impaired by HCC. It could be inferred the insufficient glucose content of DCs could not be able to afford enough energy to perform their physiological functions, such as migration in peripheral tissue and interaction with naive T cells in lymph node. This could explain why the overall number of DCs reaching a lymph node is very small (probably less than 1%) after their intracutaneous injection into a host loading tumor [6,7]. In addition, the insufficient energy supplies caused by tumor cells -derived factors might affect the other functions of DCs, such as the metabolisms of protein and lipid, this inevitably might be associated with the uptake, processing and presenting of antigen of DCs. It was one of the possible reasons of the deteriorated motilities and immune regulatory functions of DCs under tumor microenvironment.

Taken together, the present study suggested that gene transcriptional activity and energy states of DCs were inhibited by HCCs, this might be one of aspects of tumor immune escape. The ratios of absorption intensity of FT-IR at given wave number were closely correlated with the expression levels of NF-κB. It laid the foundation for the application of FT-IR to the identification of functional states in the DCBV preparation protocol.

## Abbreviations

DCs: Dendritic cells; imDCs: Immature dendritic cell; mDCs: Mature dendritic cells; DCBV: Dendritic cells-based vaccination; FTIR: Fourier transformed infrared spectroscopy; NF-κb: Nuclear factors-kappa B; HCCs: Hepatocellular carcinoma cell line; HUVECs: Human umbilical vein endothelial cells; HC: Normal hepatocyte cell line; ASA: Aspirin.

## Competing interests

The authors declare that they have no competing interests.

## Authors’ contributions

ZZ: composed the manuscript, RD, JL and CZ: analyzed the data and worked on the methods, ZW and LL: proposed the idea, WY: made the discussions. All authors read and approved the final manuscript.
